# Microvesicles released from fat-laden cells promote activation of hepatocellular NLRP3 inflammasome: A pro-inflammatory link between lipotoxicity and non-alcoholic steatohepatitis

**DOI:** 10.1371/journal.pone.0172575

**Published:** 2017-03-01

**Authors:** Stefania Cannito, Elisabetta Morello, Claudia Bocca, Beatrice Foglia, Elisa Benetti, Erica Novo, Fausto Chiazza, Mara Rogazzo, Roberto Fantozzi, Davide Povero, Salvatore Sutti, Elisabetta Bugianesi, Ariel E. Feldstein, Emanuele Albano, Massimo Collino, Maurizio Parola

**Affiliations:** 1 Department of Clinical and Biological Sciences, University of Torino, Torino, Italy; 2 Department of Drug Science and Technology, University of Torino, Torino, Italy; 3 Department of Pediatrics, University of California San Diego (UCSD), La Jolla, CA, United States of America; 4 Department of Health Sciences and Interdisciplinary Research Center for Autoimmune Diseases, University “Amedeo Avogadro” of East Piedmont, Novara, Italy; 5 Department of Medical Sciences, Division of Gastroenterology, University of Torino, Torino, Italy; Medizinische Fakultat der RWTH Aachen, GERMANY

## Abstract

Non-Alcoholic Fatty Liver Disease (NAFLD) is a major form of chronic liver disease in the general population in relation to its high prevalence among overweight/obese individuals and patients with diabetes type II or metabolic syndrome. NAFLD can progress to steatohepatitis (NASH), fibrosis and cirrhosis and end-stage of liver disease but mechanisms involved are still incompletely characterized. Within the mechanisms proposed to mediate the progression of NAFLD, lipotoxicity is believed to play a major role. In the present study we provide data suggesting that microvesicles (MVs) released by fat-laden cells undergoing lipotoxicity can activate NLRP3 inflammasome following internalization by either cells of hepatocellular origin or macrophages. Inflammasome activation involves NF-kB-mediated up-regulation of NLRP3, pro-caspase-1 and pro-Interleukin-1, then inflammasome complex formation and Caspase-1 activation leading finally to an increased release of IL-1β. Since the release of MVs from lipotoxic cells and the activation of NLRP3 inflammasome have been reported to occur in vivo in either clinical or experimental NASH, these data suggest a novel rational link between lipotoxicity and increased inflammatory response.

## Introduction

Non-Alcoholic Fatty Liver Disease (NAFLD) has emerged in recent years as a major form of chronic liver disease affecting both children and adults worldwide, with a prevalence ranging from 3–15% in the general population and up to 70% among overweight individuals [1–5]. Epidemiological data indicate that 20–30% of NAFLD patients, particularly obese and/or diabetic type II and/or those affected by metabolic syndrome, can develop Non-Alcoholic Steato-Hepatitis (NASH) and fibrosis and eventually progress to cirrhosis and end-stage liver disease [1–9]. In the natural history of the disease, an increase in hepatic lipid deposit (i.e., fatty liver or steatosis) is considered a required early event and prerequisite, potentially benign, for the development of NASH [1–9]. Along these lines, a large body of literature data supports the notion that upon lipid accumulation within parenchymal cells certain lipids, in particular saturated fatty acids, can exert cyto-toxic effects also known as lipotoxicity, resulting in hepatocyte damage and in triggering inflammatory responses [10–12].

In this scenario, recent data suggest that fat-laden hepatocytes undergoing lipotoxicity may release extracellular vesicles (EVs). EVs are an heterogeneous family of small membrane vesicles released by dying or activated cells that includes exosomes (30–100 nm in diameter), released by exocytosis and microparticles or microvesicles (MVs, 100–1000 nm in diameter) [13,14]. MVs, in particular, are small vesicles surrounded by a phospholipid bilayer, generated and released through a controlled budding/blebbing of the plasma membrane [13]. These MVs can act in an autocrine/paracrine manner carrying to surrounding cells several molecules, including surface receptors, membrane, cytosolic or even nuclear proteins, lipids and RNAs (mRNAs and microRNAs) [14–16]. These MVs, can either remain in the tissue of origin or get into the blood circulation, delivering “molecular information” to target cells by either interacting with surface receptors and/or following internalization [17–18].

Concerning liver parenchymal cells, previous studies have established that both primary hepatocytes and immortalized cells of hepatocellular origin can release both exosomes and MVs [19–22]. Furthermore, increased circulating levels of MVs are associated with liver injury in either “in vivo” models of chronic liver diseases or human blood samples from patients with NAFLD and alcohol or chronic hepatitis C related cirrhosis [19, 22–26]. With regard to NAFLD progression, we have reported that MVs are released by hepatocytes undergoing lipotoxicity in a caspase-3 dependent manner and act as pro-angiogenic and profibrogenic stimuli promoting endothelial and hepatic stellate cells activation [22,23]. In the same experimental setting a recent study has also shown that MVs released by fat-laden hepatocytes or HuH7 cells may act as pro-inflammatory stimuli on macrophages through signals operated by tumor necrosis factor-related apoptosis-inducing ligand (TRAIL), expressed on the surface of these MVs [27]. Along these lines, one of the most recently identified contributor to the cross talk between hepatocytes and inflammatory macrophages is represented by the multiprotein platform complex nucleotide-binding oligomerization domain-like receptor protein 3 (NLRP3) inflammasome, whose activation has been suggested to play a crucial role in the progression of NAFLD [28]. In particular, the transition from NAFLD to NASH associates with NLRP3-inflammasome activation and an increased expression of inflammasome-related components, including apoptosis-associated speck-like protein containing a carboxy-terminal CARD (ASC), caspase-1 (CASP‑1) and pannexin [28–32]. In addition, inflammasome activation involves not only liver innate immunity cells but also parenchymal cells [29,31,32], with studies indicating that saturated fatty acids can specifically activate the inflammasome complex in hepatocytes inducing IL-1β expression and release [29,32]. However, no study has so far investigated whether MVs released from fat-laden hepatocytes may promote NLRP3-inflammasome activation [28].

In the present study we provide for the first time evidence that MVs released by fat-laden cells can directly up-regulate NLRP3 inflammasome in both hepatocytes and macrophages, resulting in a significant increase in IL‑1β release.

## Materials and methods

### Materials

Enhanced chemiluminescence (ECL) reagents, nitrocellulose membranes (Hybond-C extra), and secondary Cy3-conjugated antibodies were from Amersham Pharmacia Biotech Inc. (Piscataway, NJ, USA). Polyclonal antibodies against NF-kB p65 (#8242), IKKβ (#2370) and p-IKK α/β (#2697) or monoclonal antibodies against IKB (#4814) and p-IKB (#9246) were from Cell Signalling Technologies (Danvers, Massachusetts); polyclonal antibodies against IL-1β (sc-7884), caspase p10 (sc-515) and LaminA (sc-20680) were from Santa Cruz Biotechnology (Santa Cruz, CA, USA); monoclonal antibodies against NLRP3 were from Abcam (Cambridge, UK, code ab109314) or from Adipogen (Adipogen AG, Liestal, Switzerland, code AG-20B-0014-C100). Monoclonal antibodies for α-tubulin and β-actin and all other reagents of analytical grade were from Sigma Chemical Co (Sigma Aldrich Spa, Milan, Italy). HiPerfect Transfection Reagent was from Qiagen (QIAGEN S.r.l., Milan, Italy). ELISA kit for human IL-1β was from Abcam (Cambridge, UK, code ab100562).

### Animal experiments

Eight-week old male C57BL/6 mice were purchased from Charles River Laboratories (Charles River UK Ltd., Margate, UK) and fed for 4 or 8 weeks with either methionine- and choline-deficient (MCD) diet or related control diet sufficient in methionine and choline (MCS) (Laboratorio Dottori Piccioni, Gessate, Italy) as previously described [33]. Experiments were approved by the Italian Ministry of Health and by the university commission or animal care following the criteria of the Italian National Research Council. The experimental MCD and MCS diets were also administered to C57BL/6 caspase 3 knockout (Casp3−/−) mice and related wild type littermates up to 6 weeks exactly as previously described [22]. The latter genetically manipulated mice and their littermates were treated in compliance with the Guide for the Care and Use of Laboratory Animals (National Academy of Science,Washington, DC), and animal procedures were approved by the University of California, San Diego, Institutional Animal Care and Use Committee.

### Cell lines and culture conditions

HepG2 and THP-1 cells (American Type Culture Collection, USA) were maintained in Dulbecco’s modified Eagle’s medium or RPMI 1640 respectively supplemented with 10% fetal-bovine serum, 100 U/ml penicillin, 100 μg/ml streptomycin and 25 μg/ml amphotericin-B. To evaluate the role of LPS, palmitic acid (PA, 0,25mM) or MVs, HepG2 cells were seeded in normoxic conditions to obtain the desired sub-confluence level (65–70%) and then incubated in experimental conditions (up to 48h hours). THP-1 cells were seeded 7x10^5^ in 35 mm petri dishes and differentiated for 48 hrs with phorbol 12-myristate 13-acetate (PMA, 50nM); after 24 hrs of incubation with fresh medium THP-1 cells were properly stimulated. mRNA, cellular extracts and the corresponding medium were tested for inflammasome components (NLRP-3 and caspase-1) and related cytokine (IL-1β). In some experiments HepG2 cells were transfected with specific siRNA for caspase-3 or NLRP3 and non-silencing siRNA (scramble, SC).

### MVs isolation and purification and other related methodological issues

The procedures adopted in this study to isolate, purify, quantify and characterize MVs obtained from fat-laden HepG2 have been extensively described in previous studies [22,23,26]. Briefly, for MVs isolation HepG2 cells were seeded onto a 100-mm dish and cultured until they reached 80 to 85% confluency. Cells were then incubated with 0.25 mM palmitic acid (PA) in serum-free DMEM, supplemented with 1.1% penicillin and streptomycin and 1% endotoxin-free bovine serum albumin (BSA) for up to 24 hours. The uptake of FFAs from hepatocytes was evaluated with an Oil Red-O staining according to the manufacturer’s instruction (see later). Control cells were incubated with the same serum-free medium supplemented with the vehicle used to dissolve PA.

MVs were then isolated by differential centrifugation and characterized essentially as previously described [22,23,26]. The isolation procedure involved the following steps: i) collected media were centrifuged at 3000 g for 15 min to remove cell debris and aggregates; ii) the supernatant from last centrifugation were then transferred to new tubes and ultra-centrifuged at 100,000 g for 90 minutes at 10° C; iii) the supernatants collected from the latter step were used as MVs-free control whereas the pelleted MVs fraction were gently re-suspended into 500 μl of PBS for flow cytometry or in 500 μl of serum-free DMEM medium and used for subsequent in vitro studies. To trace hepatocyte-derived MVs in flow cytometry and confocal analysis, the PKH26 fluorescent dye (Sigma-Aldrich) was used, according to the manufacturer’s instructions and as previously described (22).

A complete characterization of MVs, including size, composition and distribution, was performed by dynamic light scattering, TEM (see here S1A Fig) and FACS as reported previously [22].

MVs were quantified either by the bicinchoninic acid (BCA) protein assay [22] or (see S1B Fig) by flow cytometry using MVs resuspended in PBS and the Calcein AM (BD Biosciences, San Jose, CA, USA)—based procedure previously described [26]. The preparations of MVs employed in the present study were also tested for presence of NLRP3 and IL-1β protein levels and, as shown in S1C Fig, we could not find any trace of such proteins in pelleted MVs. In addition, no trace of mRNA levels for NLRP3 or IL-1β could be detected in MVs (data not shown).

### Quantitative real-time PCR (Q-PCR)

RNA extraction, complementary DNA synthesis, quantitative real-time PCR (Q-PCR) reactions were performed as previously described [34]. Human or murine NLRP3, IL1β and CASP-1 mRNA levels were measured by Q-PCR, using the SYBR® green method as described [34]. The amplification mix was prepared using Roche LightCycler FastStart DNA MasterPLUS SYBR Green I kit following manufacturer’s instructions and real-time PCR was performed using LightCycler instrument. Oligonucleotide sequence of primers used for RT-PCR were: sense, 5’-TGAAAGCTCTCCACCTCCAG-3’, reverse 5’-CACGCAGGACAGGTACAGAT-3’ (for human IL-1β); sense, 5’-AAGGAAGTGGACTGCGAGAA-3’, reverse 5’-CCCTCGAATTTGCCATA-3’ (for human NLRP3); sense, 5’-GCTTTCTGCTCTTCCACACC-3’, reverse 5’-CATCTGGCTGCTCAAATGAA-3’ (for human CASP-1); sense, 5'-TGCTCTTCACTGCTATCAAGCCCT-3', reverse 5'-ACAAGCCTTTGCTCCAGACCCTAT-3′ (for murine NLRP3); sense, 5'-GGAGAACCAAGCAACGACAAAATA-3', reverse 5'-TGGGGAACTCTGCAGACTCAAAC-3′ (for murine IL-1β); sense, 5′-TCCGCGGTTGAATCCTTTTCAGA-3', reverse 5′-ACCACAATTGCTGTGTGTGCGCA-3 (for murine caspase-1); sense, 5'-TGGCCCAGACCCTCACACTCAG-3', reverse, 5'-ACCCATCGGCTGGCACCACT-3' (for murine TNF-α). Gliceraldehyde-3-phosphate dehydrogenase (GAPDH) was used as internal reference and co-amplified with target samples using identical Q-PCR conditions. Samples were run in triplicate and mRNA expression was generated for each sample. Specificity of the amplified PCR products was determined by melting curve analysis and confirmed by agarose gel electrophoresis.

### Caspase-3 and NLRP3 silencing by small RNA interference

RNA interference experiments to knockdown caspase-3 or NLRP3 expression in HepG2 cells were performed using siRNA duplex (Qiagen Italia, Milano, Italy) as previously described [35]. The following target sequences were used:

NLRP3: 5’-AAGGTGTTGGAATTAGACAAC-3’;
CASP3: 5’-CTGAGATGGGTTTATGTATAA-3’.

The siRNAs and related non-silencing controls were transfected in HepG2 cells with HiPerfect Transfection reagent (Qiagen Italia, Milano, Italy) according to manufacturer’s instructions up to 48hrs (CASP3) or 72hrs (NLRP3). Transfected cells in fresh medium were then exposed to the desired experimental conditions and then harvested for sample preparation.

### Western blot analysis

Total cell lysates and nuclear vs cytosolic extracts, obtained as described, [35, 36] as well as MVs preparations (the latter by resuspending pelleted MVs directly in RIPA buffer) were subjected to sodium dodecyl sulfate-polyacrylamide gel-electrophoresis on 12%, 10% or 7.5% acrylamide gels, incubated with desired primary antibodies, then with peroxidase-conjugated anti-mouse or anti-rabbit immunoglobulins in Tris-buffered saline-Tween containing 2% (w/v) non-fat dry milk and finally developed with the ECL reagents according to manufacturer’s instructions. Sample loading was evaluated by reblotting the same membrane with antibodies raised against β-actin or α-tubulin or, for nuclear extracts, Lamin A.

### Immunohistochemistry, immunofluorescence and histo-morphometric analysis

Paraffin liver sections of specimens derived from patients with NAFLD/NASH-related advanced fibrosis or derived from either wild type C57BL/6 mice or caspase 3 ^-/-^ mice fed MCD diet or related control diet (MCS) as well as from the liver of NLRP3 ^+/-^ mice, were analyzed as previously described [33,34]. The use of human material conforms to the ethical guidelines of the 1975 Declaration of Helsinki and was approved for this study by the University of Torino Bioethical Committee. Immunostaining procedure were performed, according to established procedures previously described in detail [33,37], on paraffin sections (2 μm thick), mounted on poly-L-lysine coated slides, that were incubated with the antibodies against NLRP3 (Abcam, Cambridge, UK, dilution 1:100, or from Adipogen AG, Liestal, Switzerland, dilution 1:100) and murine F4/80 (Affimetrix, Cleveland, OH, USA, dilution 1:1000) or human CD68 (Bio-Rad; Segrate—MI, dilution 1:40). After blocking endogenous peroxidase activity with 3% hydrogen peroxide and performing microwave antigen retrieval, primary antibodies were labeled by using EnVision, HRP-labeled System (DAKO) and visualized by 3’-diaminobenzidine substrate. For negative controls the primary antibodies were replaced by isotype- and concentrations-matched irrelevant antibody [33,37]. Quantification of NLRP3 positive immunostaining in murine liver was also performed by histo-morphometric analysis using a digital camera and a bright field microscope to collect images that were then analyzed by employing the ImageJ software.

Indirect immunofluorescence and confocal microscopy analysis was performed on cultured cells as previously described [35,36] by employing a primary rabbit polyclonal antibody raised against NF-kB p65 (Cell Signalling Technology, Danvers, MA, USA; diluted 1:400). Nuclei were stained using 4,6-diamino-2-phenylindole (DAPI, blue fluorescence).

### Internalization of MVs

Cultured cells, seeded in 6-well culture plates (10^5^ cells/well) or on microscope slides, were exposed for 1 hour (THP-1) or 3 hrs (HepG2) to PKH26 fluorescent dye -MVs released by HepG2 treated with palmitic acid (0,25mM) as previously described [22]. Internalization of MVs was evaluated by: a) flow cytometric analysis: cells were rapidly washed with PBS, collected by trypsinization and re-suspended in PBS for analysis. Detection of PKH26 red fluorescent die-MVs (FL2) was performed on at least 5,000 cells per sample with a FACScan using the CellQuest software (Becton-Dickinson, Milano, Italy); b) indirect immunofluorescence and confocal microscopy analysis: nuclei (blue fluorescence) were stained using 4,6-diamino-2-phenylindole (DAPI), MVs were stained with PKH26 fluorescent die (red fluorescence) and cytoskeleton were marked with Alexa Fluor 488 phalloidin (high-affinity filamentous actin, F-actin, green fluorescence) and slides were examined with an Olympus Fluoview 300 confocal laser scanning microscope.

### Red oil staining

HepG2 cells were seeded in 6-wells culture plates until reaching 80–85% of confluence and incubated with 0.25 mM palmitic acid in serum-free DMEM, supplemented with 1.1% penicillin and streptomycin and 1% endotoxin-free BSA, for up to 24 hours. Control cells were incubated with the same serum-free medium supplemented with the vehicle used to dissolve the FFAs. After treatment, cells were fixed with 4% of paraformaldehyde and the uptake of FFAs from HepG2 cells was detected by staining cells for 30 minutes with Oil-Red-O 0,5% (Sigma-Aldrich, St. Louis, MO, USA) diluted in isopropanol, according to the manufacturer's instruction. Then, bright field microscope was employed to collect images.

### Biochemical analysis

Plasma alanine aminotransferase (ALT) and liver triglycerides (TGs) were determined by spectrometric kits supplied by Radim S.p.A. (Pomezia, Italy) and Sigma Diagnostics (Milano, Italy), respectively. Release of IL-1β in culture medium was evaluated, according to the manufacturer’s instructions by commercial enzyme-linked immunosorbent assay (ELISA) kit supplied by Abcam (Cambridge, UK).

### Detection of caspase-3/7 activity in cell culture

Caspase-3/7 activity as a measure of apoptotic cell death was determined in HepG2 cells exposed to palmitic acid (0,25 mM) for 24 hrs and following the manufacturer’s instructions (Apo-ONE Caspase-3/7 Homogeneous Assay, (Promega, Madison, WL, USA). After the treatment HepG2 cells were washed twice with PBS and a volume of 50 μL of homogeneous caspase-3/7 reagent was added to each well. The plate was incubated for 1 h at room temperature. After this time, fluorescence was measured in a fluorimeter at excitation 499 nm and emission 521 nm.

### MTT assay

Viability of HepG2 cells was evaluated by the MTT colorimetric assay. HepG2 cells were seeded 1x10^4^ in 96-wells plates for each wells and incubated with 0.25 mM palmitic acid in serum-free DMEM, supplemented with 1.1% penicillin and streptomycin and 1% endotoxin-free BSA, for up to 24 hours. Control cells were incubated with the same serum-free medium supplemented with the vehicle used to dissolve the FFAs. After treatment, 30 μl of 3-(4,5-dimethylthiazol-2-yl)-2,5-diphenyltetrazolium bromide (MTT, 5mg/ml in PBS solution) was added to each well and incubated up to 2 hrs at 37°. Then, 150 μl of DMSO was added to empty wells for 20 minutes at room temperature. After this time, fluorescence was measured in a ELISA plate reader at absorbance of 540 nm.

### Statistical analysis

For cell culture experiments data in bar graphs represent means ± SEM, and were obtained from average data of at least three independent experiments. Luminograms and morphological images are representative of at least three experiments with similar results. Statistical analysis for these experiments was performed by Student’s t-test or ANOVA for analysis of variance when appropriate (p < 0.05 was considered significant).

## Results

**Fig 1:**
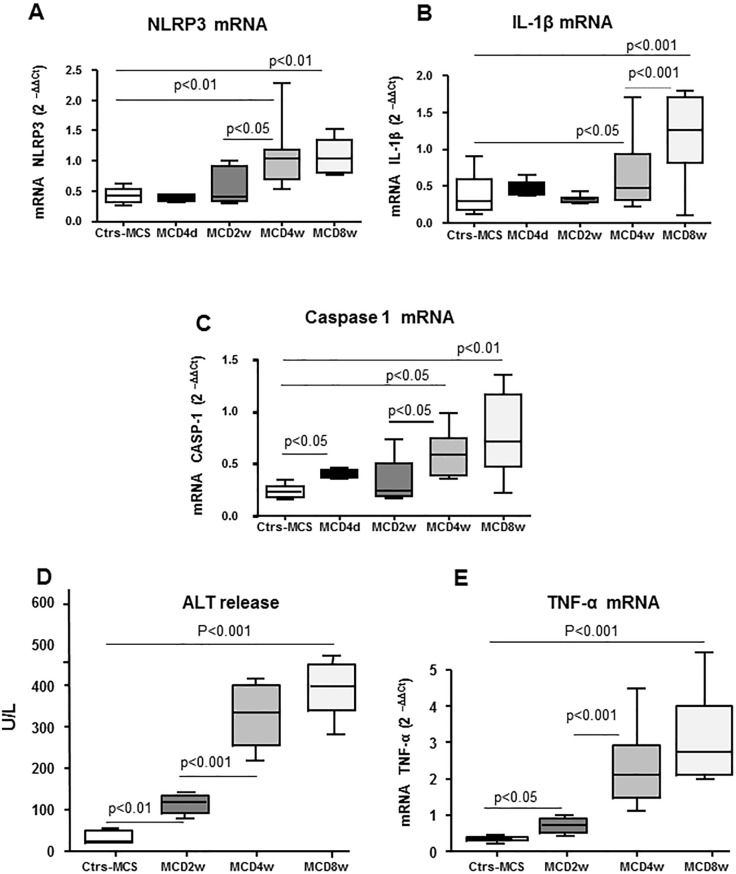
In vivo time-dependent analyses of inflammasome components, inflammatory cytokines and necrosis in MCD fed mice. “In vivo” analysis by quantitative real-time PCR (qPCR) of transcripts of inflammasome components (NLRP3 and CASP-1 **A, C**) and related cytokine (IL-1β, **B**) as well as of TNF-α (**E**) in WT mice fed with MCS diet or MCD diet for 4d, 2wks, 4wks and 8wks. **D**. Analysis of serum ALT in WT mice fed with MCS diet or MCD diet for 2wks, 4wks and 8 wks. Data in graphs are expressed as means ± SEM (n = 6 mice for any experimental group); p values are indicated and referred to mice fed the MCS control diet.

The aim of the present study has been to investigate the possible role of MVs released from fat-laden cells in promoting NLRP3 inflammasome activation in either parenchymal cells and macrophages. According to recent literature data, inflammasome activation is fully detectable in association with overt steatohepatitis in experimental murine models of progressive NASH and such an activation involves liver innate immunity cells and hepatocytes [29,31,32]. In line with these findings, our experiments using mice fed with a methionine/choline deficient (MCD) diet showed that the transcript levels for NLRP3 and IL1β were significantly up-regulated after 4 weeks of treatment (Fig 1A, Fig 1B) in parallel with an appreciable worsening of lobular inflammation and parenchymal damage (Fig 1D, Fig 1E). No appreciable changes were evident at earlier time points. Similar data were obtained by evaluating caspase 1 transcripts, with the exception of a transient increase detected at 4 days (Fig 1C).

**Fig 2:**
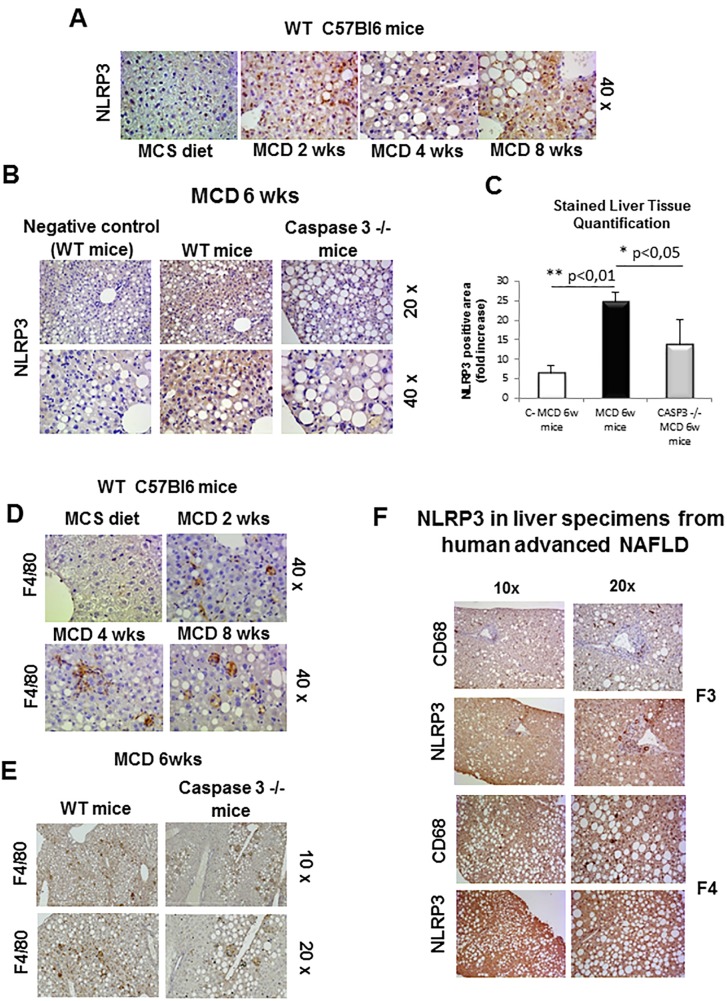
Immunohistochemistry analysis for NLRP3 and F4/80. **A,B.** Immunohistochemistry analysis for NLRP3 on liver specimens from WT mice fed with MCS diet or MCD diet for 2wks, 4wks and 8wks (**A**) as well as WT mice and Casp 3^-/-^ knockout mice fed for 6wks (**B**). **C.** Image analysis quantification for NLRP3 staining (Fig 2B) as evaluated with Image*j* software in liver sections from WT or caspase 3 -/- mice fed a MCD diet for 6 weeks. **D,E.** Immunohistochemistry analysis for F4/80 on liver specimens from WT mice fed with MCS diet or MCD diet for 2wks, 4wks and 8wks (**D**) as well as WT mice and Casp 3^-/-^ knockout mice fed for 6wks (**E**). **F**. Immunohistochemistry analysis for NLRP3 and CD68 on liver specimens from human NAFLD/NASH-related advanced fibrosis (F3 and F4). Original magnification as indicated.

In line with these data, NLRP3 immunohistochemical analysis showed and appreciable increase in mice fed the MCD diet for 4 weeks (Fig 2A). In these animals, NLPR3 up-regulation paralleled with a progressive recruitment of hepatic macrophages as evidenced by F4/80 immunostaining (Fig 2D). This scenario is also consistent to what is occurring in liver specimens from human NASH in which NLRP3 positivity was detectable at immunohistochemistry in parenchymal cells and in CD68 positive macrophages (Fig 2F).

Since in a previous study some of us reported that MVs production associated with NASH required caspase-3 activity and was appreciably decreased in caspase-3 knock out (caspase-3^-/-^) mice fed with the MCD diet [22], here we evaluated whether the lack of MVs production in caspase-3^-/-^ animals might affect NLRP3 responses. As shown in Fig 2B, NLRP3 staining was appreciably reduced in caspase-3^-/-^ mice as compared to WT littermates, as also confirmed by histo-morphometric analysis (Fig 2C). In a similar manner liver macrophage infiltration was significantly lower in MCD-fed caspase-3^-/-^ mice as compared to similarly treated WT animals (Fig 2E).

These data were confirmed by histo-morphometric analysis by employing a different antibody targeting NLRP3 either in NLRP3 ^+/-^ mice (S2A Fig) as well in either wild type and Caspase 3 ^-/-^ mice fed 6 wks with MCD diet (S2B Fig).

On the basis of these observations, we then designed experiments to directly investigate the hypothesis that MVs released by fat-laden cells may affect synthesis and activity of NLRP3 inflammasome. In order to do so we employed a well established “in vitro” model to generate MVs based on the exposure of hepatocytes or immortalized cells (HepG2 or HuH7) cells to 0.25 mM palmitic acid [22,27]. In the present study, in order to maintain reproducible conditions in the different experiments, we employed human HepG2 cells.

**Fig 3:**
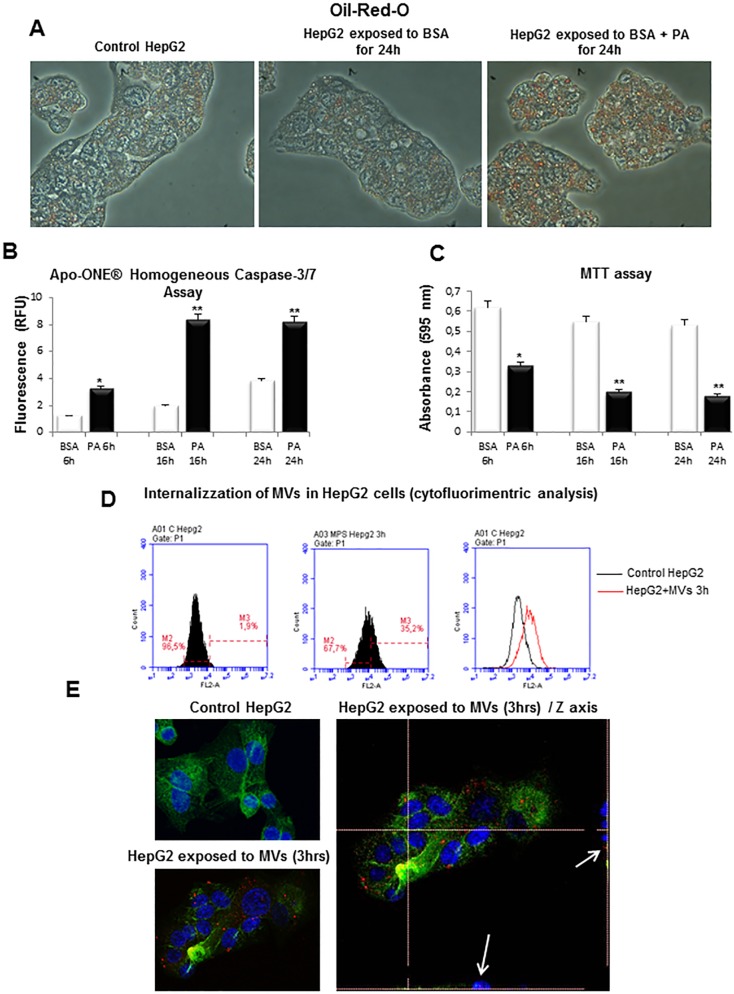
In vitro experimental model of lipotoxicity. **A.** Red Oil-O staining in control HepG2 cells, HepG2 cells treated with BSA 1% or HepG2 cells exposed to palmitic acid 0.25mM in BSA 1% (BSA + PA) for 24hrs. **B.** Detection of Caspase-3/7 Activity in HepG2 cells treated with BSA 1% or PA 0.25mM for indicated times, analyzed by using Apo-ONE Caspase-3/7 Homogeneous Assay. **C.** Viability of HepG2 cells treated with BSA 1% or PA 0.25mM for indicated times, evaluated by MTT assay. **D,E.** Analysis of internalization of MVs in HepG2 cells by (**D**) flow cytometry or by (**E**) confocal microscopy: nuclei (blue fluorescence), MVs (red fluorescence) and cytoskeleton (F-actin, green fluorescence).

As expected, the incubation of HepG2 cells with palmitic acid resulted in a significant intracellular accumulation of lipids, as shown by Oil-Red-O staining (Fig 3A), as well as in a conditions of progressive caspase–dependent lipoapoptosis, as evidenced by Caspase 3/7 and MTT assays (Fig 3B, Fig 3C). The culture media was used to isolate MVs released by fat laden HepG2-cells as previously described [22]. Protein content of the preparations of MVs in these experiments gave mean routine values from control cells of 0.02–0.03 μg/μl vs values of 4.3–4.6 μg/μl from cells exposed to PA for 24 hrs.

Interestingly, the addition to HepG2 cells of purified MVs conjugated with the fluorescent dye PKH26 lead to an internalization (within 3 hours) of red fluorescent MVs as shown by either flow cytometry analysis (Fig 3D) or by using confocal laser microscopy (Fig 3E).

**Fig 4:**
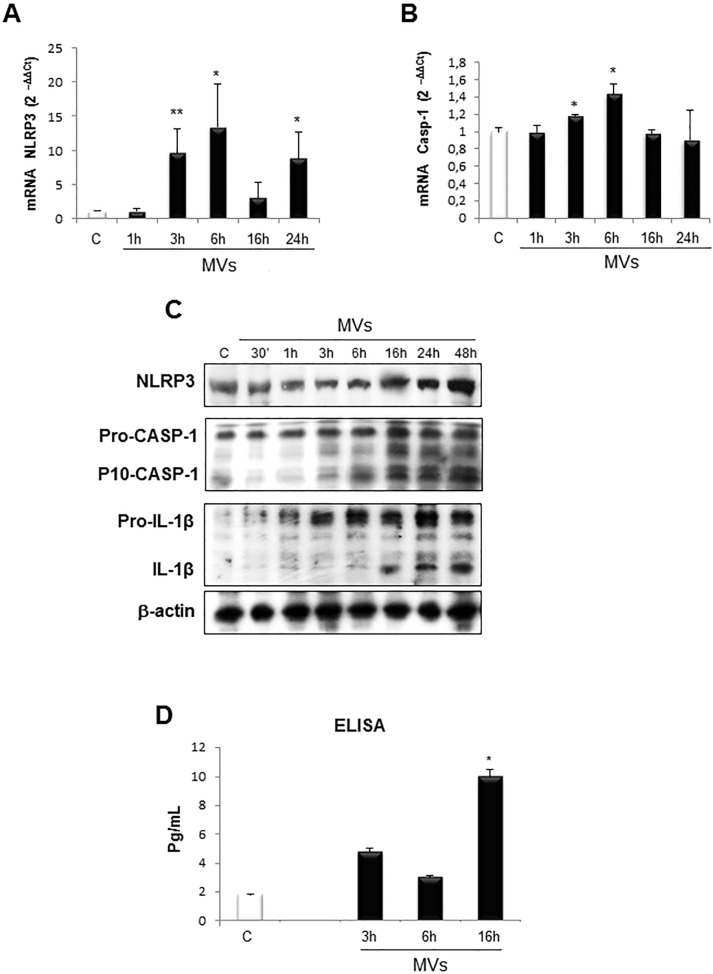
MVs activate NLRP3 inflammasome cascade. **A, B.** Quantitative real-time PCR (qPCR) analysis of inflammasome components (NLRP3 and CASP-1) in HepG2 naïve cells treated with MVs up to 24 hrs. **C.** Western blot analysis of activation of inflammasome pathway by evaluating NLRP3 protein levels as well as cleaved caspase-1 (p-10-CASP-1) and IL-1β in total extracts obtained by HepG2 naïve cells exposed to MVs for indicated times. Equal loading was confirm by re-probing the same membrane with monoclonal antibody against the house-keeping β-actin. **D.** ELISA assay to evaluate IL-1β release (pg/ml) in culture medium of HepG2 naïve cells exposed to MVs up to 16 hrs. Data in graphs are expressed as means ± SEM (*p< 0.05 and ** p<0.01 vs related control HepG2 cells) of three independent experiments.

We next analysed whether HepG2 cells exposure to MVs may modulate inflammasome activation. Fig 4 shows that MVs addition to resting HepG2 cells was followed within 3 hours by a significant up-regulation of the transcripts levels for NLRP3 (Fig 4A) and Caspase-1 (Fig 4B). When analysed in Western blots MVs-treated HepG2 cells also showed time-dependent increase in NLRP3, pro-IL-1β and pro-caspase-1 proteins levels that were associated with an early detection of active caspase-1 that was detected starting from 3 hrs and detectable until 48 hrs (Fig 4C). Conversely IL-1β protein levels were consistently enhanced at 16 hrs and thereafter (Fig 4C). ELISA assay analysis of IL-1β released in the culture media revealed that MVs exposure resulted in a rather early, although limited, release of the cytokine at 3 and 6 hrs to reach a significant level at 16 hrs (Fig 4D).

**Fig 5:**
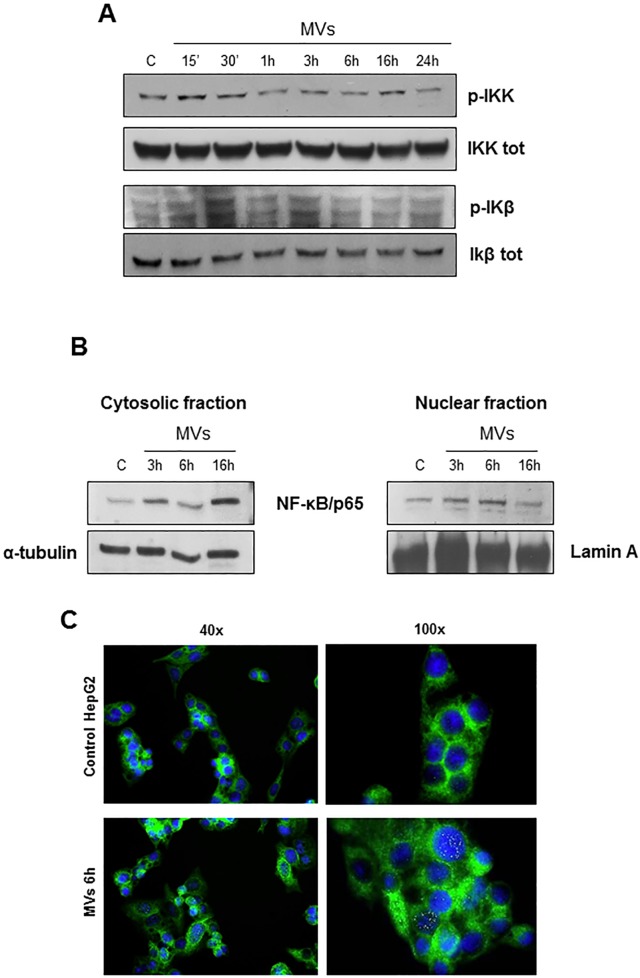
MVs up-regulate NF-KB pathway. **A.** Western blot analysis of activation of IKK and IKβ evaluated in total extracts of HepG2 naïve cells exposed to MVs for indicating time. Equal loading was confirmed by re-probing the same membrane with un-phosphorylated protein. **B**. Western blot analysis of NF-Kb/p65 protein levels in cytosolic and nuclear extract obtained from HepG2 naïve cells exposed to MVs up to 16 hrs. Equal loading was confirmed by re-probing membrane with α-tubulin and Lamin-A (cytosolic and nuclear house-keeping, respectively). **C**. Immunofluorescence analysis of NF-Kb/p65 nuclear translocation in control HepG2 cells or HepG2 cells exposed to MVs for 6hrs: nuclei (blue, DAPI staining), NF-Kb/p65 (green). Original magnifications are indicated.

It is known that NLRP3 expression requires NF-kB phosphorylation and its nuclear translocation [28]. To get more insights on the mechanisms responsible for inflammasome activation by MVs we next analysed whether MVs may be able to modulate NF-kB pathway. In our hand, HepG2 cells exposure to MVs was followed by a very early phosphorylation of both IkB kinase (IKK) and of Ikβ (Fig 5A) as well as by an increase of p65 subunit in both the cytosolic and in nuclear extracts (Fig 5B). Nuclear translocation of p65 was further confirmed by means of indirect immunofluorescence (Fig 5C).

**Fig 6:**
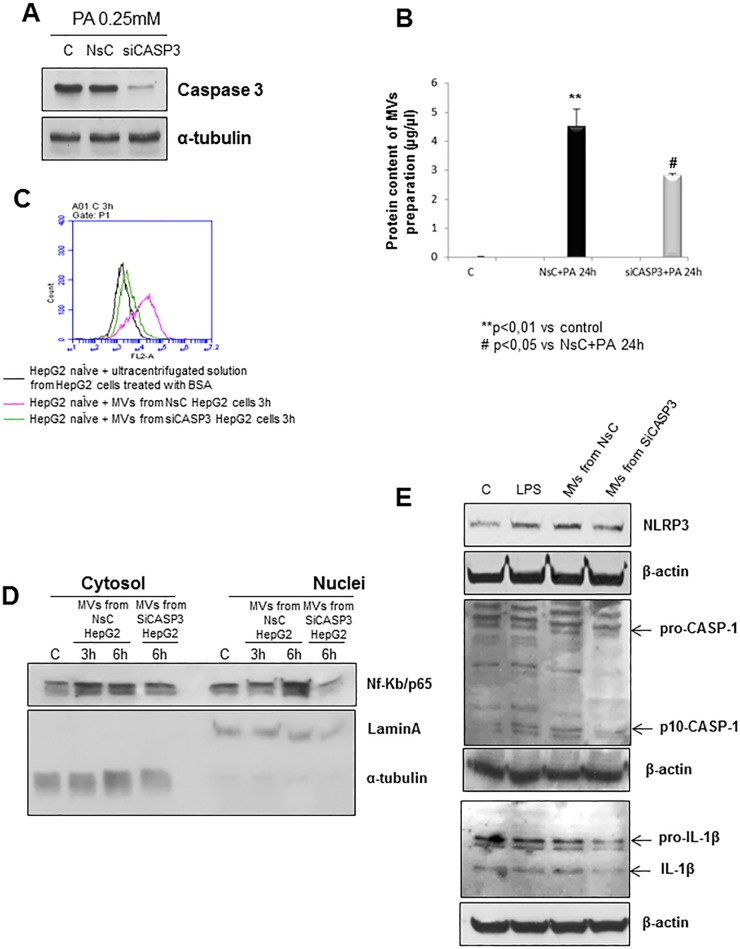
NLRP3 inflammasome activation is prevented by blocking the caspase 3 –dependent release of MVs. **A.** Western blot analysis of caspase-3 in total extracts of HepG2 cells not transfected (C), transfected with non-silencing siRNA (NsC) or specific siRNA for caspase-3 (siCASP3) and treated with palmitic acid 0,25mM for 24hrs. Equal loading was confirm by re- probing the same membrane with monoclonal antibody against the house-keeping β-actin. **B.** Protein quantification of preparation of MVs as obtained from control HepG2 cells (C), from HepG2 cells transfected with the non silencing RNA and then exposed to PA (NsC) and HepG2 cells silenced for caspase 3 and then exposed to PA (siCASP3). Data are expressed as μg/μl and as mean ± SD being (n = 12 for any condition). **C.** Flow cytometric analysis of internalization of PKH26-fluorescent die-MVs in HepG2 naïve cells. Color legend: i) black trace is related to HepG2 naïve cells treated with ultracentrifugated solution (also marked with PKH26-fluorescent die) derived from cells just exposed to BSA (used as control), ii) pink trace is related to HepG2 naïve cells treated with MVs derived from HepG2 cells transfected with non-silencing treated with palmitic acid 0,25mM for 24hrs; iii) green trace is related to HepG2 naïve cells treated with MVs derived from HepG2 cells transfected with specific siRNA for caspase-3 and treated with palmitic acid 0,25mM for 24hrs. **D.** Western blot analysis of NF-Kb/p65 protein levels in cytosolic and nuclear extract obtained from HepG2 naïve cells exposed or not (C) to MVs derived from HepG2 cells transfected with non-silencing siRNA (NsC) or specific siRNA for caspase-3 (siCASP3) and treated with palmitic acid 0,25mM for 24hrs. Equal loading was confirmed by re-probing membrane with α-tubulin and Lamin-A (cytosolic and nuclear house-keeping, respectively). **E.** Western blot analysis of NLRP3 protein levels as well as of cleaved caspase-1 (p-10-CASP-1) and IL-1β in total extracts obtained by HepG2 naïve cells exposed or not (C) to MVs derived from HepG2 cells transfected with non-silencing siRNA (NsC) or specific siRNA for caspase-3 (siCASP3) and treated with palmitic acid 0,25mM for 24hrs. LPS 1mg/ml was used as positive control. Equal loading was confirmed by re-probing membrane with β-actin.

From the data showing that caspase-3 deficiency can prevent NLRP3 up-regulation in mice fed the MCD diet, we next decided to deplete this enzyme by means of a specific siRNA. According to previous reports [22], blocking caspase-3 in HepG2 cells before lipid loading (Fig 6A) resulted in a decrease in the release of MVs that (as also documented by analysis of protein content of MVs preparations from cells silenced or not for caspase 3, see Fig 6B) that was reflected by a sharp reduction in MVs internalization in naïve HepG2 cells (Fig 6C) as compared to cells exposed to non-silencing siRNA. Moreover, the relationships between MVs and NF-kB pathway was further supported by the observation that the exposure of naïve HepG2 cells to MVs from cells treated with caspase-3 siRNA resulted in a significant decrease in p65 nuclear translocation as compared to cells receiving MVs from cells treated with the non-silencing vector (Fig 6D). Accordingly, silencing of caspase-3 in the parental fat-laden cells led to a decrease in the protein levels of the inflammasome components (Fig 6E) as compared to fat-laden cells receiving the non-silencing control vector.

**Fig 7:**
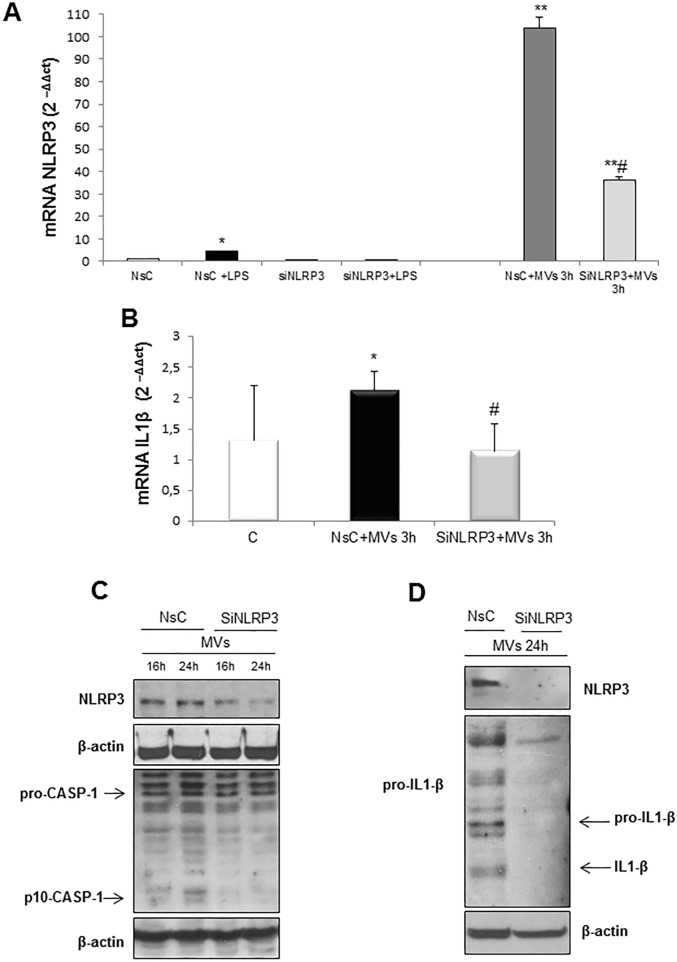
Silencing of NLRP3 in target cells prevents MVs-dependent inflammasome activation. **A.** Quantitative real-time PCR (qPCR) of NLRP3 mRNA in HepG2 cells transfected for 72hrs with non-silencing siRNA (NsC) or specific siRNA for NLRP3 (siNLRP3) and exposed or not to MVs derived from HepG2 cells treated with PA 0.25mM for 24hrs. LPS 1mg/ml was used as positive control. **B.** Quantitative real-time PCR (qPCR) of IL-1β mRNA in HepG2 cells not transfected (C) and transfected for 72hrs with non-silencing siRNA (NsC) or specific siRNA for NLRP3 (siNLRP3) and exposed or not for 3hrs to MVs derived from HepG2 cells treated with PA 0.25mM for 24hrs. Data in graphs are expressed as means ± SEM (*p< 0.05 and ** p<0.01 vs related control HepG2 cells; # p<0.05 vs NsC+MVs) of three independent experiments. **C,D.** Western blot analysis of NLRP3 protein levels as well as of cleaved caspase-1 (p-10-CASP-1) and IL-1β in total extracts obtained by HepG2 cells transfected for 72hrs with non-silencing siRNA (NsC) or specific siRNA for NLRP3 (siNLRP3) and exposed for indicating time to MVs derived from HepG2 cells treated with PA 0.25mM for 24hrs. Equal loading was confirmed by re-probing membrane with β-actin.

In order to establish whether the MVs-induced release of IL-1β was directly dependent on NLRP3 inflammasome, HepG2 cells were depleted of NLRP3 using a specific siRNA before exposure to MVs for 16 or 24 hrs. In control arm cells were exposed to a non-silencing vector. HepG2 cells silenced for NLRP3 responded to MVs by a sharp decrease in NLRP3 transcript (Fig 7A) as well as by low protein levels of activated caspase-1 (Fig 7B) and IL-1β (Fig 7C). In addition, NLRP3 silencing in HepG2 cells also resulted (Fig 7D) in a reduction of transcript levels for IL-1β (i.e., pro-IL-1β in this case).

**Fig 8:**
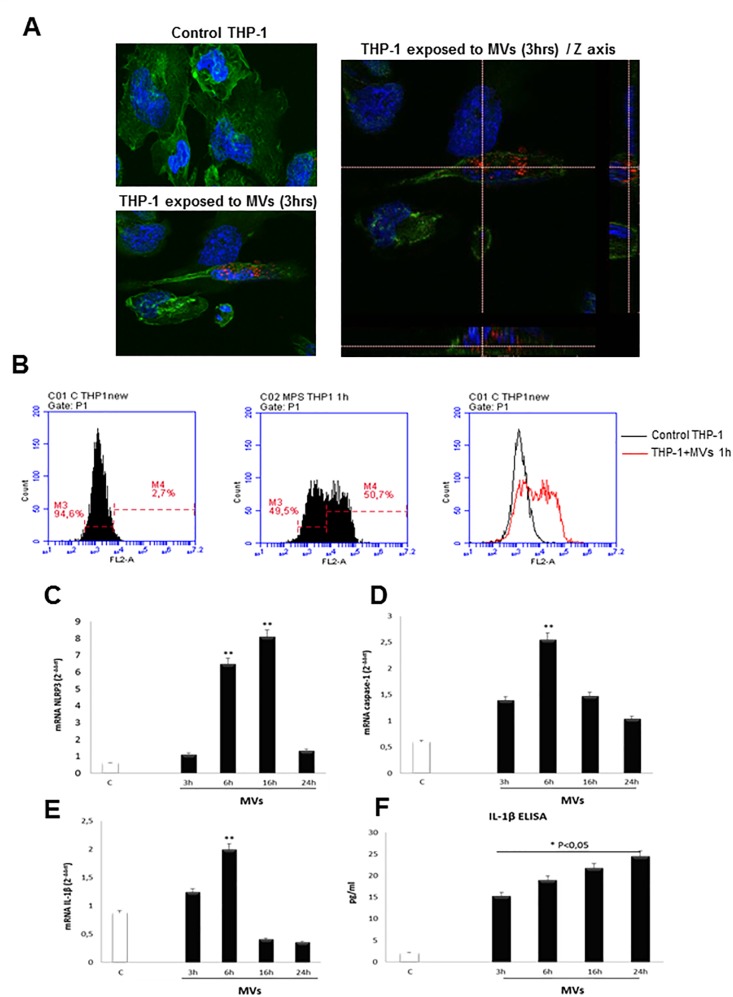
MVs activate NLRP3 inflammasome in THP-1 differentiate macrophages. **A,B.** Analysis of internalization of MVs in THP-1 cells by confocal microscope (3hrs): nuclei (blue fluorescence), MVs (red fluorescence) and cytoskeleton (F-actin, green fluorescence) (**A**) or by flow cytometry (1 hr, **B**). **C-E**. Quantitative real-time PCR (qPCR) of NLRP3, caspase-1 and IL-1β mRNAs in THP-1 cells treated with MVs derived from HepG2 cells treated with PA 0.25mM for 24hrs, for indicating times. Data in graphs are expressed as means ± SEM (*p< 0.05 and ** p<0.01 vs related control THP-1 cells). **F**. ELISA assay to evaluate IL-1β release (pg/ml) in culture medium of THP-1 differentiated macrophages exposed to MVs up to 24 hrs. Data in graphs are expressed as means ± SEM (*p< 0.05 and ** p<0.01 vs related control HepG2 cells) of three independent experiments.

Finally, additional experiments were performed to verify whether MVs may be able also to promote inflammasome activation in macrophages. To this aim, we exposed macrophage differentiated from human THP-1 cells to MVs obtained from fat-laden HepG2 cells. In differentiated THP1 cells we observed a rapid (as early as after 1 hr) internalization of MVs marked with the fluorescent dye PKH26 (Fig 8A, Fig 8B) as well as an increase in transcript levels of NLRP3, Caspase-1 and IL-1β (Fig 8C–8E) accompanied by a time-dependent increase in IL-1β release in the extracellular medium (Fig 8F).

## Discussion

Hepatocyte injury due to lipotoxicity and inflammatory infiltration by cells of innate immunity, mainly activated macrophages, are two well recognized pathological hallmarks of NASH [10–12,38]. In this scenario, multiple hits involving oxidative stress, endotoxemia, cytokines and gut microbiota have been suggested to play a role in favouring NAFLD progression to NASH [10–12,38–40]. In recent years different reports have provided evidence that during NAFLD progression hepatocyte injury induced by lipotoxicity may affect, and somehow drive, the response of surrounding cells through the release of hepatocyte-derived MVs. At present, MVs released by lipotoxic hepatocytes have been proposed to up-regulate angiogenic and pro-fibrogenic responses in endothelial cells [19,22] and hepatic stellate cells [23], respectively. Moreover, lipotoxicity-mediated MVs release has been recently shown to initiate a CHOP/DR5/caspase 8/caspase-3 signalling cascade leading to the release of MVs expressing on their surface TRAIL as molecule able to trigger a pro-inflammatory and NF-kB–dependent response in macrophages [27], thus acting act as “messengers” between injured hepatocytes and macrophages [27,41].

In the present study we provide novel evidence suggesting that MVs released by cells undergoing lipotoxicity may actively contribute to pro-inflammatory responses by activating in a paracrine way NLRP3 inflammasome in either parenchymal cells and macrophages with a mechanism that involves MVs internalization by “target” cells. This is in agreement with previous data showing that endocytosis is required for the triggering of MVs responses in endothelial and hepatic stellate cells [22,23]. The inflammasome stimulation results in caspase-1 activation and a sustained release of IL-1β, an event that can support the lobular inflammation in NASH and contributes to the maintenance of insulin resistance [42–47].

It is well known that the inflammasome represents a multimeric protein complex which senses several stimuli related to pathogen and damage-associated molecular patterns in tissues undergoing infections, cell damage or metabolic imbalances [28,48,49]. The formation of the inflammasome complex leads to the activation of caspase-1, which in turn proteolytically cleaves the precursors of pro-inflammatory cytokines IL-1β and IL-18. Inflammasome responses can also cause pyroptosis, a peculiar and rapid pro-inflammatory form of cell death relevant in the so-called “sterile inflammation” which is known to occur also in NASH [49]. Along these lines, a recent study using mice expressing a constitutively active form of NLRP3 under the control of the endogenous NLPR3 promoter, has shown that increased NLRP3 activity results in extensive liver inflammation, hepatocyte pyroptosis, and liver fibrosis [30]. Indeed, activation of NLRP3 inflammasome in both hepatocytes and macrophages has been detected only when NAFLD had advanced to NASH [28–33,50], a scenario that seems to be confirmed by this study showing that NLRP3 protein is overexpressed in liver biopsies from NASH patients. Furthermore, in MCD model of NASH hepatic NLRP3 and IL-1β transcript levels are both increased only in severe steatohepatitis.

To characterize the mechanisms involved in MVs—induced activation of NLRP3 inflammasome we used a well characterized “in vitro” model of MVs release by fat-laden cells. This model has been used by different laboratories in recent years [22,23,27] and is based on the exposure of HepG2 cells to palmitic acid resulting in rapid intracellular lipid accumulation that leads to the induction of caspase 3 –dependent lipoapoptosis and the release of MVs. The capacity of MVs released by cells undergoing lipotoxicity to activate NLRP3 inflammasome is supported by data indicating that activation is prevented by either blocking MVs production by silencing caspase 3 in lipotoxic cells before exposure to palmitate or by the silencing NLRP3 target cells before exposure to MVs. Furthermore, MVs promote target cells release of IL-1β in the extracellular medium. Such a release is evident in both HepG2 cells and macrophages 16 hrs after the addition of MVs, but already detectable, although at lower levels, at earlier time points. The analysis of the events triggered by MVs suggests that they induce an early transcription of NLRP3 and pro-IL-1β, likely through NF-kB–dependent signals as recently described by Hirsova et al. [27]. However, protein analysis shows that that NLRP3 and pro-caspase 1 are already present in resting cells and that their apparent levels increase not before 6 hrs of exposure to MVs. Of interest, the levels of active caspase 1 that is responsible for the cleavage of pro-IL-1β start to increase already 1 hr after exposure to MVs. This suggests that MVs internalization may lead to the assembly and activation of NLRP3 inflammasome even before an increase in NLRP3 and pro-caspase 1 protein levels. This interpretation is in agreement with literature data stating that NLRP3 canonical inflammasome activation can occur as a two-step process, in which a first signal up-regulates NLRP3 expression through NFκB-dependent pathways [51], while a second signal leads to multimeric protein complex assembly and activation through distinct pathways [52–54], that involve the interaction between the danger signal(s) and the NACHT domain of NLRP3. Although in the present study we have not yet characterized the precise nature of signal(s) leading to MVs-mediate assembly/activation of inflammasome, the overall message is of potential relevance and further supports the concept that MVs released by damaged cells undergoing lipotoxicity can have a role in sustaining hepatic inflammation in NASH along with previously shown fibrogenesis and angiogenesis. Further experimental studies are needed in order to fully clarify and detail the reported relationships between the release of MVs from fat laden hepatocytes and surrounding cells in the scenario of NAFLD/NASH chronically injured microenvironment.

## Supporting information

S1 Fig Characterization of microvesicles (MVs) obtained from fat-laden HepG2 cells. **(A).** Representative TEM micrograph of HepG2-derived MVs released after 24 hrs of 0.25 mM palmitic acid (PA) treatment. Scale bar 200 nm. (**B)** Flow cytometry analysis (Whisker plot) of Calcein+ MVs per mL of media isolated from fat-laden HepG2 cells at the end of 24 hrs exposure to PA. Values represent mean ± SD of 6 different MVs preparations. *P < 0.05. **(C).** Western blot analysis of NLRP3 and IL-1β in the following experimental condition: 1: total extract from MVs release from HepG2 cells treated for 24hrs with BSA (C-); 2: total extract from MVs release from HepG2 cells treated for 24hrs with PA 25mM; 3:total extract from control HepG2 cells; 4: total extract from HepG2 cells treated with LPS 1μg/μl for 1 hr; 5: total extract from HepG2 cells treated with PA 25mM for 24hrs. Equal loading was evaluated by re-probing the same membrane with the monoclonal antibody targeting β-actin. Red ponceau images were also included to show protein loading.
(TIF)

S2 Fig Immunohistochemistry analysis for NLRP3 on liver specimens from NLRP3 hemizygous +/-mice (**A**) as well as WT mice or Casp 3-/- knockout mice fed for 6wks with MCD diet (**B**). Original magnification as indicated. Right panels represent histomorphometric analysis (ImageJ program) that has been performed on n = 4 liver sections obtained from three different animals for each condition indicated in panels A and B in order to evaluate positive staining for NLRP3.
(TIF)

## Abbreviations

ASC: apoptosis-associated speck-like protein containing a carboxy-terminal CARD
BDL: bile duct ligation
BSA: bovine serum albumin
CASP-1: caspase 1
CASP3: caspase 3
DMSO: dimethyl-sulfoxide
ECL: enhanced chemiluminescence
EVs: extracellular vesicles
IL‑1β: interleukin‑1β
LPS: lipopolysaccharide
MCD diet: methionine-choline deficient diet
MCS diet: methionine-choline sufficient diet
MVs: microvesicles
NAFLD: non-alcoholic fatty liver disease
NASH: non-alcoholic steatohepatitis
NF-kB: nuclear factor kB
NLRP3: nucleotide-binding oligomerization domain-like receptor protein 3
qPCR: quantitative real-time PCR
PA: palmitic acid
TRAIL: tumor necrosis factor-related apoptosis-inducing ligand
T2DM: Type 2 diabetes mellitus
