# Supplementary figures and images for: Microvesicles released from fat-laden cells promote activation of hepatocellular NLRP3 inflammasome: A pro-inflammatory link between lipotoxicity and non-alcoholic steatohepatitis
Source: PLoS One. 2017 Mar 1;12(3):e0172575. doi: 10.1371/journal.pone.0172575 (PMC5331985; doi:10.1371/journal.pone.0172575)

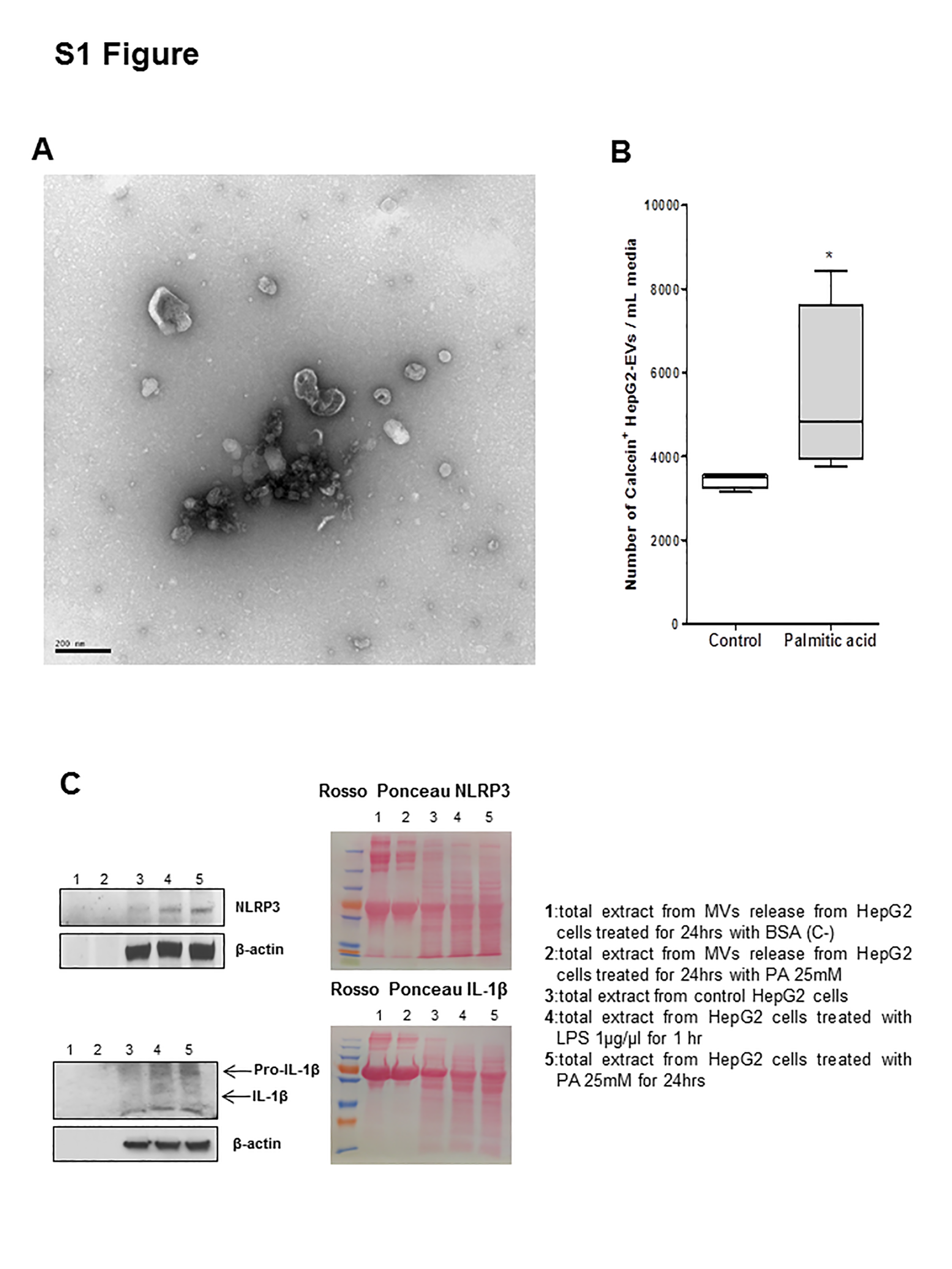

Supplement: S1 Fig — (A). Representative TEM micrograph of HepG2-derived MVs released after 24 hrs of 0.25 mM palmitic acid (PA) treatment. Scale bar 200 nm. (B) Flow cytometry analysis (Whisker plot) of Calcein+ MVs per mL of media isolated from fat-laden HepG2 cells at the end of 24 hrs exposure to PA. Values represent mean ± SD of 6 different MVs preparations. *P < 0.05. (C). Western blot analysis of NLRP3 and IL-1β in the following experimental condition: 1: total extract from MVs release from HepG2 cells treated for 24hrs with BSA (C-); 2: total extract from MVs release from HepG2 cells treated for 24hrs with PA 25mM; 3:total extract from control HepG2 cells; 4: total extract from HepG2 cells treated with LPS 1μg/μl for 1 hr; 5: total extract from HepG2 cells treated with PA 25mM for 24hrs. Equal loading was evaluated by re-probing the same membrane with the monoclonal antibody targeting β-actin. Red ponceau images were also included to show protein loading. (TIF) [file pone.0172575.s001.TIF]

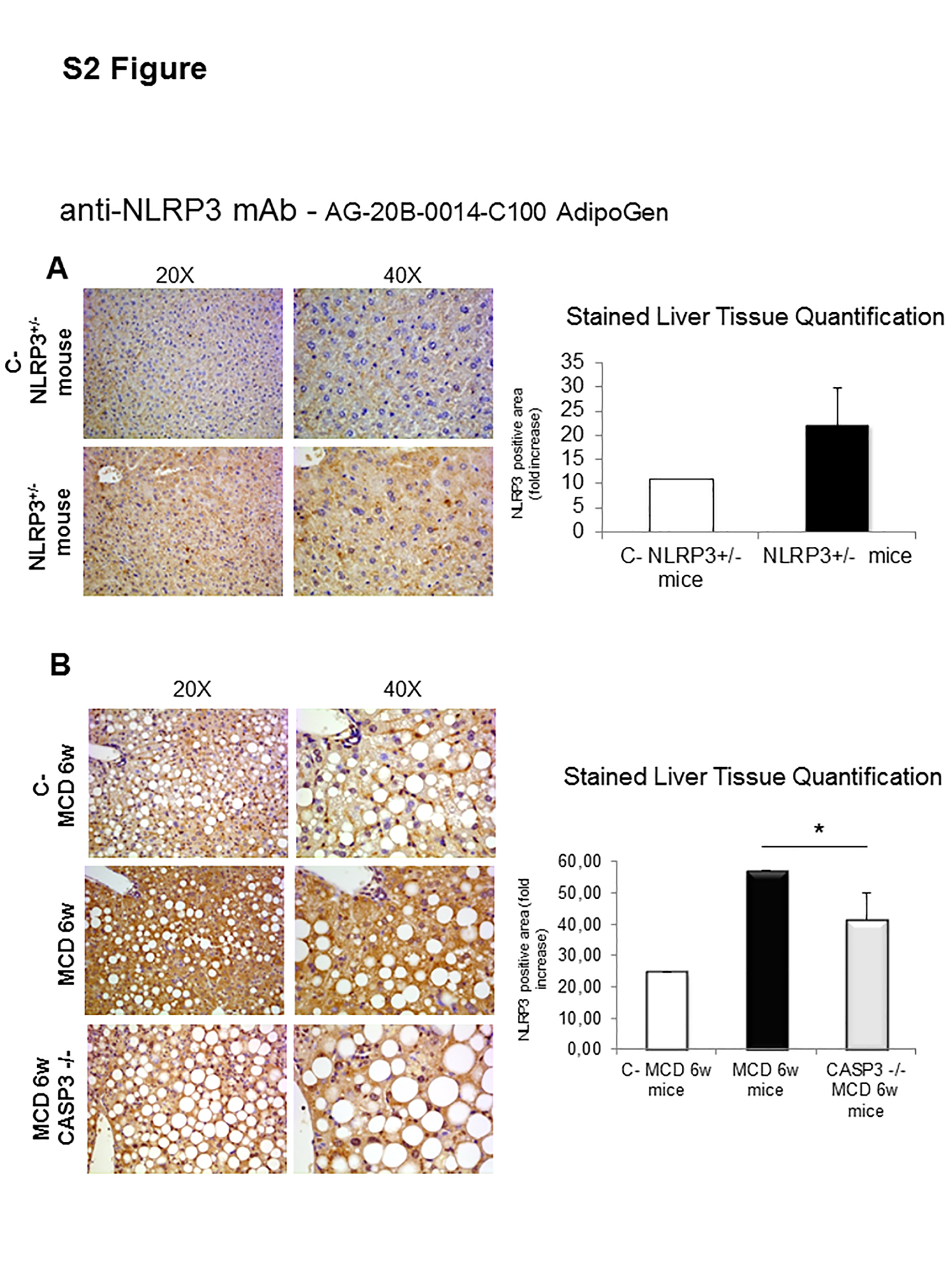

Supplement: S2 Fig — Immunohistochemistry analysis for NLRP3 on liver specimens from NLRP3 hemizygous +/-mice (A) as well as WT mice or Casp 3-/- knockout mice fed for 6wks with MCD diet (B). Original magnification as indicated. Right panels represent histomorphometric analysis (ImageJ program) that has been performed on n = 4 liver sections obtained from three different animals for each condition indicated in panels A and B in order to evaluate positive staining for NLRP3. (TIF) [file pone.0172575.s002.TIF]
